# Effect of Plasma On-Time with a Fixed Duty Ratio on Reactive Species in Plasma-Treated Medium and Its Significance in Biological Applications

**DOI:** 10.3390/ijms24065289

**Published:** 2023-03-09

**Authors:** Sohail Mumtaz, Juie Nahushkumar Rana, Jun Sup Lim, Rida Javed, Eun Ha Choi, Ihn Han

**Affiliations:** 1 Department of Electrical and Biological Physics, Kwangwoon University, Seoul 01897, Republic of Korea; sohail.ahmed2015@gmail.com (S.M.); junsup117@gmail.com (J.S.L.); ehchoi@kw.ac.kr (E.H.C.); 2 Plasma Bioscience Research Center (PBRC), Applied Plasma Medicine Center, Kwangwoon University, Seoul 01897, Republic of Korea; ranajuie06@gmail.com (J.N.R.); ridajaved.uaf@gmail.com (R.J.); 3 Department of Plasma Bio-Display, Kwangwoon University, Seoul 01897, Republic of Korea

**Keywords:** nonthermal atmospheric pressure plasma, duty ratio, plasma on-time, ROS and RNS, plasma treated medium, cold plasma

## Abstract

Optimizing the therapeutic range of nonthermal atmospheric pressure plasma (NTAPP) for biomedical applications is an active research topic. For the first time, we examined the effect of plasma on-times in this study while keeping the duty ratio and treatment time fixed. We have evaluated the electrical, optical, and soft jet properties for two different duty ratios of 10% and 36%, using the plasma on-times of 25, 50, 75, and 100 ms. Furthermore, the influence of plasma on-time on reactive oxygen and nitrogen species (ROS/RNS) levels in plasma treated medium (PTM) was also investigated. Following treatment, the characteristics of (DMEM media) and PTM (pH, EC, and ORP) were also examined. While EC and ORP rose by raising plasma on-time, pH remained unchanged. Finally, the PTM was used to observe the cell viability and ATP levels in U87-MG brain cancer cells. We found it interesting that, by increasing the plasma on-time, the levels of ROS/RNS dramatically increased in PTM and significantly affected the viability and ATP levels of the U87-MG cell line. The results of this study provide a significant indication of advancement by introducing the optimization of plasma on-time to increase the efficacy of the soft plasma jet for biomedical applications.

## 1. Introduction

An ionized gas made up of charged particles (electrons and ions), radicals, excited atoms and molecules, and visible and UV photons is referred to as plasma. This intricate combination has applications across a variety of fields [1,2]. Nonthermal atmospheric pressure plasma (NTAPP) is a recently developed technology that can be used for a number of purposes, such as plasma medicine, agriculture, and disinfection against microbial bacteria and fungi. It is currently used in particular for the disinfection of viruses against COVID-19 [2,3,4,5]. Additionally, it is frequently used to whiten teeth and inactivate oral microorganisms [6,7], as well as to promote faster wound healing [8]. Disinfection of microbial using NTAPP could be achieved by interactions between the plasma reactive oxygen and reactive nitrogen species (ROS/RNS) and soft materials in cell membrane protein, RNA, or DNA. Most plasmas are generated in the gaseous state using air, helium, neon, argon, nitrogen molecules, and their mixtures according to the required purposes.

For decades, NTAPP was used to treat different cancers and tumors [9,10,11]. It is now well known that the ROS/RNS played an important role in the biomedical application of NTAPP [12,13,14,15,16,17]. Variable anti-tumor efficacy results from differences in the composition and quantity of plasma-generated ROS/RNS between devices and from feed gas admixtures [18,19]. The NTAPP is well known for producing enormously high ROS/RNS levels. Elevated concentrations of ROS/RNS have been found to be produced by both DBD plasma and jet plasma [20,21,22]. A gas flow transports indirect plasmas generated between two electrodes of a specific device to the application region. At the point where the jet meets the surrounding air, ROS/RNS are frequently produced for a number of reasons. According to previous reports, plasma-derived ROS/RNS can cause morphological changes, membrane depolarization, lipoprotein peroxidation, and DNA damage in cells [23,24,25]. It would make sense to investigate increased ROS/RNS exposure from cold physical plasmas as anticancer agents, since these substances follow hormetic responses in biology [26]. Irrespective of whether the effects of the plasma are direct or indirect, the appropriate irradiation time needs to be considered. It has been noted that when the exposure period is very long, plasma damages both cancerous and healthy cells. However, when the exposure time is short, neither cancerous nor healthy cells are affected. Therefore, it is essential to optimize the therapeutic range of direct plasma treatment or plasma treated medium (PTM) for applications [27]. When applied to cells and tissues either directly from the plasma or by exposure to solutions that have already been plasma-treated, ROS/RNS have therapeutic effects. To maintain the low temperature in NTAPP, a duty ratio concept was proposed in the soft plasma jet [28].

Previous studies dealt with the treatment time to maximize the effect of the soft plasma jet for anticancer applications [28,29,30,31]. An important factor that needs to be resolved is the optimization of plasma on-time during the treatment time. Plasma on-time has a direct impact on RONS levels, which in turn have an impact on the biomedical effects. However, to the best of our knowledge, no studies have been conducted on the optimization of plasma on-time and its impact on the levels of RONS in PTM and the viability of cancer cells when the duty ratio and treatment time are both fixed.

In this work, we aimed to investigate the impact of plasma on-times while maintaining a fixed duty ratio and treatment time. For two different duty ratios of 10% and 36%, we have chosen the plasma on times of 25, 50, 75, and 100 ms. The preferences for ROS/RNS amounts should always be taken into consideration when choosing a soft plasma jet duty ratio. Lower duty ratios produce lower amounts of ROS/RNS, whereas higher duty ratios produce higher amounts of ROS/RNS. To compare and validate the phenomenon brought on by changing the plasma on time, we chose 10% as the low duty ratio and 36% as the high duty ratio for this study. By varying the plasma on-time with two different fixed ratios of 10% and 36%, we have evaluated the electrical and optical properties of the soft plasma jet. The properties of the PTM, including pH, electric conductivity (EC), and oxidation–reduction potential (ORP), were also investigated after treatment. Furthermore, the influence of on-time at ROS/RNS levels in PTM was also investigated to measure its concentration. Finally, the PTM treated with different plasma on-time was implemented in brain cancer cells, specifically the U87-MG line, to observe the cell viability and ATP levels. Interestingly, we observed that, by extending the plasma on-time by 25, 50, 75, and 100 ms, EC, ORP, and ROS/RNS levels significantly rose and had a significant impact on the U87 cell line’s viability and ATP levels.

## 2. Results

### 2.1. Electrical Properties of the Soft Plasma Jet

**Figure 1:**
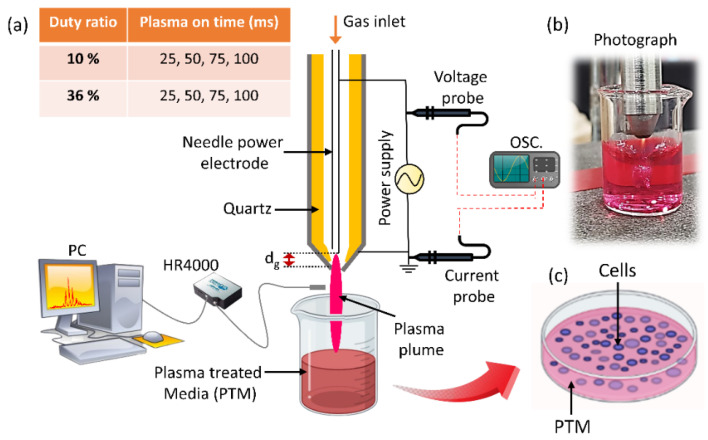
(**a**) The schematic of soft plasma jet and experimental setup. Two fixed duty ratios of 10% and 36% were used with plasma on-time (25, 50, 75, and 100 ms) to prepare PTM and its applications to the U87-MG cell line. The distance, d_g_, was used to represent the distance between the grounded electrode (nozzle) and the tip of the syringe power electrodes, which is set at 1 mm. (**b**) Photograph during treatment and (**c**) the application of PTM to the U87-MG cell line.

**Figure 2:**
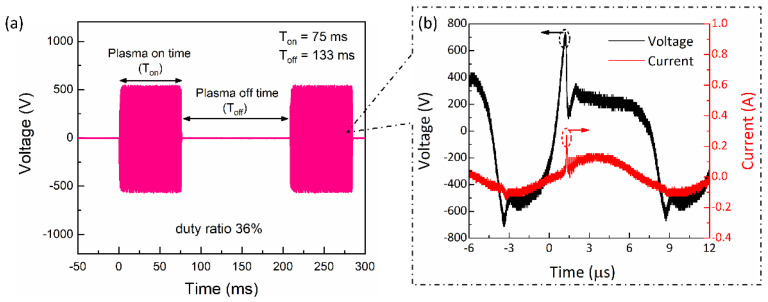
Electrical properties of discharge. (**a**) The discharge waveform shows plasma on–time (T_on_) of 75 ms and off-time (T_off_) of 133 ms with a 36% duty ratio. (**b**) A plot of the discharge current versus time in terms of current (red) and voltage (black). The current–voltage waveform was recorded when the duty ratio was 36%.

Figure 1 depicts the experimental schematic for a cold plasma jet device and how the cell medium was treated as part of the PTM preparation. The discharge waveform and current voltage waveform are shown in Figure 2a. The peak voltage and current remained essentially unchanged as the plasma-on time varied, so only one of the discharge waveforms was chosen as an example to demonstrate here. This resulted in a 36% duty ratio between the 75 ms plasma on-time (T_on_) and the 133 ms plasma off-time (T_off_). When the duty ratio was set to 36%, Figure 2b shows the typical voltage and current waveforms of plasma discharge. In this study, plasma on-times of 25, 50, 75, and 100 ms were used, with two duty ratios of 10% and 36%, as shown in Table 1. For each condition (Table 1), current–voltage and discharge waveforms with plasma on/off time were measured; the results show that there has been no appreciable change in the peak value of current and voltage. The accumulation of wall charges on the inner side of the quartz tube during discharge results in multiple discharge current peaks in the ascending portion of the discharge voltage. A few amperes of discharge current were measured over time. At the voltage waveform’s negative alternate, these charge accumulations were reversed in the negative voltage polarity, producing current peaks that resembled those in the opposite direction. According to Figure 2b, the discharge has a frequency of ~83 kHz, a peak voltage of 0.75 kV, and a peak current of 0.2 A, respectively.

The plasma on-time was maintained at 25, 50, 75, and 100 ms, with duty set at two different percentages of 10% and 36%. Table 1 lists the plasma on/off times that were chosen for the investigation in this work with duty ratios of 10% and 36%.

### 2.2. Optical Properties of the Soft Plasma Jet

**Figure 3:**
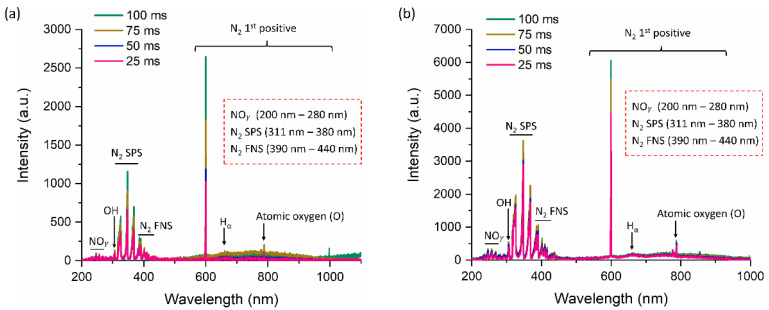
The OES spectra at plasma on-times of 25 ms, 50 ms, 75 ms, and 100 ms when the duty ratio was fixed at (**a**) 10% and (**b**) 36%.

Figure 3 displays the optical emission characteristics of the discharge NTAPP generated by a soft jet when air is a working gas. The OES of the soft jet when the duty ratio was set at 10% and the plasma on time was changed to 25, 50, 75, and 100 ms is depicted in Figure 3a. In a similar vein, Figure 3b depicts the OES for duty ratio of 36%, and plasma on-time was altered to 25, 50, 75, and 100 ms. The UV, visible, and near IR regions of the spectrum show emissions from various reactive species. The UV-C region of the spectrum, which spans 200 to 280 nm of wavelength, displayed weak emission signals from the $NO_{\gamma }$ bands [32,33,34]. The emergence of these species was attributed to the interaction of powerful electrons with the N_2_ and O_2_ molecules in the air that serves as the feeding gas [35,36,37]. By the process of UV photolysis, the UVs generated by these OH radicals also cause the production of secondary reactive species inside the liquid. Strong emissions are present in a variety of UV-B and UV-A regions [311–380 nm] from different bands of the nitrogen second positive system (N_2_ SPS). Additionally, emissions from the nitrogen first negative system (N_2_ FNS) can be seen in the visible and UV-A spectrums [390–440 nm]. At 599 nm, the first positive peak for nitrogen was also visible. These charged N_2_ species are generated when N_2_ molecules in the feeding gas and surrounding air dissociate. Additionally, the disintegration of oxygen molecules produced powerful emissions from atomic oxygen (777 nm). At 656 nm, the emission of the hydrogen-alpha line also manifested with these species. As the plasma on-time increased to 25, 50, 75, and 100 ms, the intensity of the peak increased gradually in both 10% and 36% duty ratios. The OES spectra of each condition (plasma on-time: 25, 50, 75, and 100 ms for 10% and 36% duty ratio) was plotted separately in Supplementary Figures S1 and S2 to more thoroughly observe the difference in OES intensities.

### 2.3. Effect of Plasma On-Time on the Properties of DMEM (PTM)

The properties of DMEM (PTM) were investigated after NTAPP treatment at 1 min, 3 min, 5 min, and 7 min. The temperature of the media shows a slight increase (10%: ~1–1.5 °C, 36%: ~1.5–3 °C) by increasing the treatment time to 1 min, 3 min, 5 min, and 7 min. The temperature rise is nonsignificant in PTM after treatment, as shown in Supplementary Materials Figure S3a,b.

**Figure 4:**
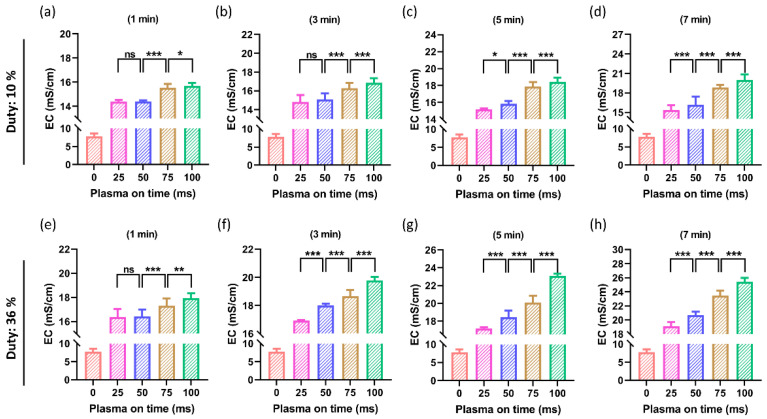
The EC of PTM (DMEM). (**a**–**d**) The EC when the duty ratio was set to 10% and (**e**–**h**) when the duty ratio of 36%. The EC of the PTM significantly increased by increasing the plasma on-time. The significance was calculated using Microsoft Excel (MS Office 365), while the differences between the treatment groups are indicated by * *p* < 0.05, ** *p* < 0.01, *** *p* < 0.001, ns—not significant.

The EC of PTM was also measured after NTAPP treatment. The EC was measured at both duty ratios of 10% and 36% by switching the plasma on-time as 25, 50, 75, and 100 ms with a treatment time as shown in Figure 4. Here, Figure 4a–d shows the obtained results when the duty ratio was fixed at 10%. The EC is depicted in Figure 4a,b for a 1 min and 3 min treatment time. All treated groups showed an increase in EC, but the difference between the increase in plasma on-time of 25 ms and 50 ms remained non-significant. When the plasma on-time was increased to 75 ms and 100 ms for a 1 min and 3 min treatment time, it was found that the EC significantly increased. At longer treatment times of 5 min and 7 min, the EC increased remarkably by increasing the plasma on-time, as shown in Figure 4c,d. Similar results are shown in Figure 4e–h when the duty ratio was set at 36%. Here, the information in Figure 4b,e is also interesting. Because higher duty ratios produce more ROS and RNS, the EC of DMEM that are attainable after a 3-min plasma treatment with a 10% duty ratio can be obtained after a 1-min plasma treatment with a 36% duty ratio. To verify this quantitatively, more research is needed.

**Figure 5:**
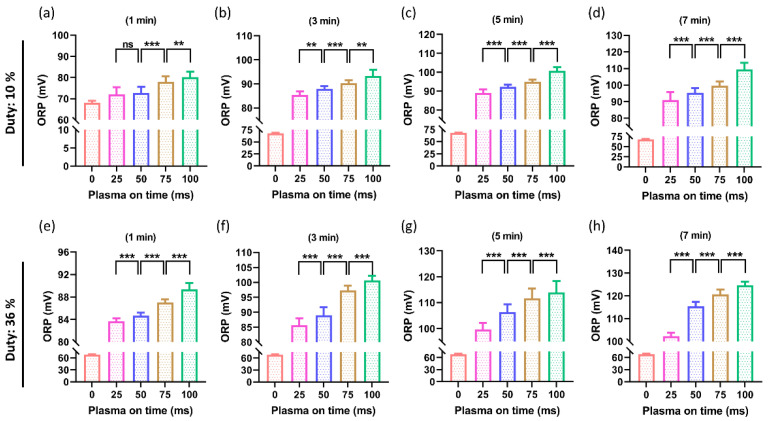
The ORP of PTM (DMEM). (**a**–**d**) The ORP when the duty ratio was set to 10% and (**e**–**h**) when the duty ratio of 36%. Increasing plasma on-time (25, 50, 75, and 100 ms) resulted in a significant increase in the ORP of the PTM. The significance was calculated using Microsoft Excel (MS Office 365), while the differences between the treatment groups are indicated by ** *p* < 0.01, *** *p* < 0.001, ns—not significant.

Following NTAPP treatment, the ORP of PTM was also assessed. By switching the plasma on-time as 25, 50, 75, and 100 ms with the selected treatment time (1, 3, 5, 7 min), the ORP was measured at both duty ratios of 10% and 36%, as shown in Figure 5. Figure 5a–d displays the outcomes when the duty ratio was set at 10%. Figure 5a shows the ORP for a 1 min treatment time. Although the ORP increased in all treated groups, there was no difference between the increases in ORP when plasm on-time was switched from 25 ms to 50 ms. The ORP was found to significantly increase when the plasma on-time was increased to 75 ms and 100 ms for a 1-min treatment time. Figure 5 illustrates how the ORP of PTM significantly increased by increasing the plasma on-time at longer treatment times of 3 min, 5 min, and 7 min (Figure 5b–d). Figure 5e–h displays comparable outcomes with a duty ratio of 36%.

**Figure 6:**
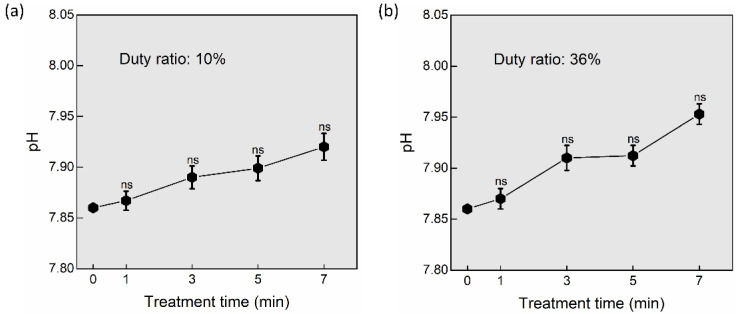
The pH of the PTM (DMEM) after plasma treatment when (**a**) 10% duty ratio and (**b**) 36% duty ratio. The pH remained unchanged or nonsignificant after a plasma treatment time of 1, 3, 5, and 7 min, ns—not significant.

The pH of DMEM was measured at treatment times (1, 3, 5, and 7 min) after NTAPP exposure to determine its impact on hydrogen ions concentration [38]. After being treated with NTAPP for 1, 3, 5, and 7 min, it was found that the pH of the DMEM remained almost unchanged (Figure 6). Additionally, by switching plasma on-time as 25, 50, 75, and 100 ms, the difference in pH was observed as non-significant or unchanged in both the 10% and 36% duty ratios (Figure 6a,b). The medium’s temperature only slightly increased by 1 to 2 degrees relative to the control and remained non-significant.

### 2.4. Effect of Plasma On-Time on ROS/RNS Levels

**Figure 7:**
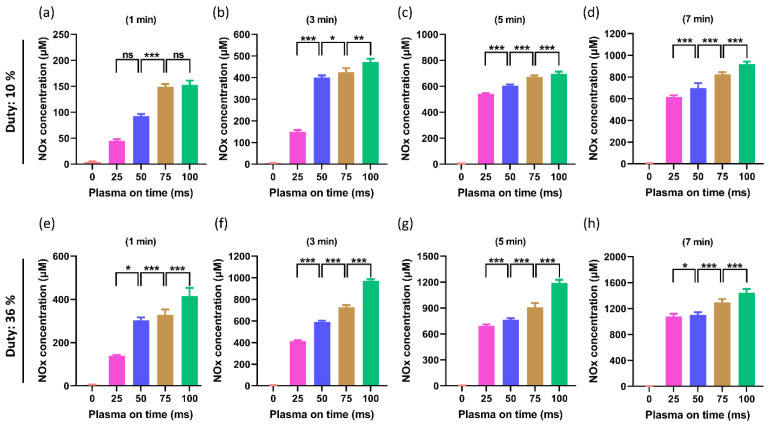
The NOx level in PTM. Only plasma on-time was altered as 25, 50, 75, and 100 ms in each treatment time and duty ratio. (**a**–**d**) The NOx content in PTM when the duty ratio was fixed at 10%. (**e**–**h**) The NOx levels when the duty ratio is maintained at 36%. It was found that the NOx levels significantly increased gradually by increasing the plasma on-time. The significance was calculated using Microsoft Excel (MS Office 365), while the differences between the treatment groups are indicated by * *p* < 0.05, ** *p* < 0.01, *** *p* < 0.001, ns—not significant.

The interaction of air plasma with DMEM activates the production of various ROS/RNS in the cell culture media (PTM), which changes the chemical composition of PTM [39,40]. Figure 7a–d shows the NOx levels in PTM when the duty ratio was 10%. A significant increase in NOx levels was observed in all treated groups of PTM when compared to the control. The purpose of this work is to determine whether altering the plasma on-time affects NOx levels when the treatment time and duty ratio are fixed. It is interesting to note that the NOx levels were significantly increased by increasing the plasma on-time as 25, 50, 75, and 100 ms with a fixed duty ratio of 10%, and results were shown in Figure 7a–d. Similar to this, a higher duty ratio of 36% was also tested to observe the NOx levels in order to confirm this effect. Figure 7e,f illustrate the effects of increasing the plasma on-time to 25, 50, 75, and 100 ms, with the same treatment time and fixed duty ratio of 36% on the NO_x_ levels.

**Figure 8:**
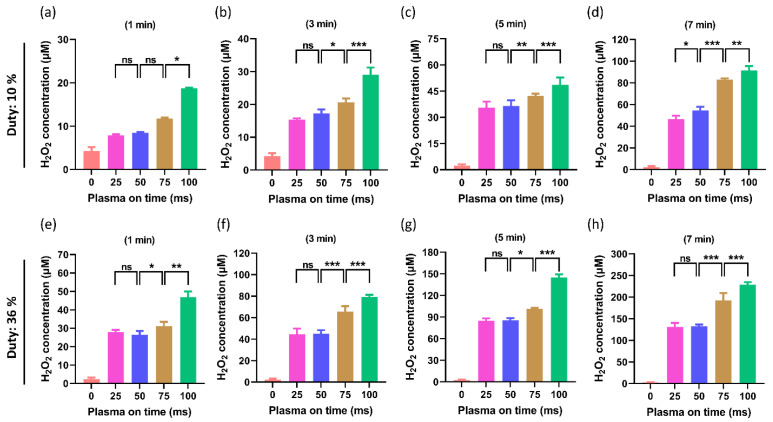
The H_2_O_2_ content in PTM. Only plasma on-time was altered as 25, 50, 75, and 100 ms in each treatment time and duty ratio. (**a**–**d**) The H_2_O_2_ content in PTM when the duty ratio was fixed at 10%. (**e**–**h**) The H_2_O_2_ when the duty ratio is maintained at 36%. It was found that the H_2_O_2_ levels significantly increased gradually by increasing the plasma on-time. The 25 ms and 50 ms plasma on-time showed similar H_2_O_2_ levels. The significance was calculated using Microsoft Excel (MS Office 365), while the differences between the treatment groups are indicated by * *p* < 0.05, ** *p* < 0.01, *** *p* < 0.001, ns—not significant.

The H_2_O_2_ levels in PTM at a 10% duty ratio are shown in Figure 8a–d. When compared to the control, all PTM-treated groups showed a significant rise in H_2_O_2_ levels (non-treated). This research seeks to determine whether changing the plasma on-time has an impact on H_2_O_2_ levels when the treatment time and duty ratio are fixed. It is interesting to note that, when the plasma on time was changed from 25 ms to 50 ms, the H_2_O_2_ levels remained non-significant. When the plasma on time increased at 75 ms and 100 ms, the increase in H_2_O_2_ was significant, and this phenomenon remained in all treatment times (1, 3, 5, and 7 min). In order to confirm this effect, a duty ratio test with a higher duty rate of 36% was also conducted. The effects of increasing the plasma on-time at 25, 50, 75, and 100 ms, with the same treatment time and fixed duty ratio of 36% on the levels of H_2_O_2_, are shown in Figure 8e–h. It is interesting to note that, when the plasma on-time changed from 25 ms to 50 ms, the H_2_O_2_ levels remained insignificant. When the plasma on-time increased at 75 ms and 100 ms, the increase in H_2_O_2_ levels was noticeable.

### 2.5. The Influence of the Plasma On-Time on the Viability of the U87-MG Cell Line

**Figure 9:**
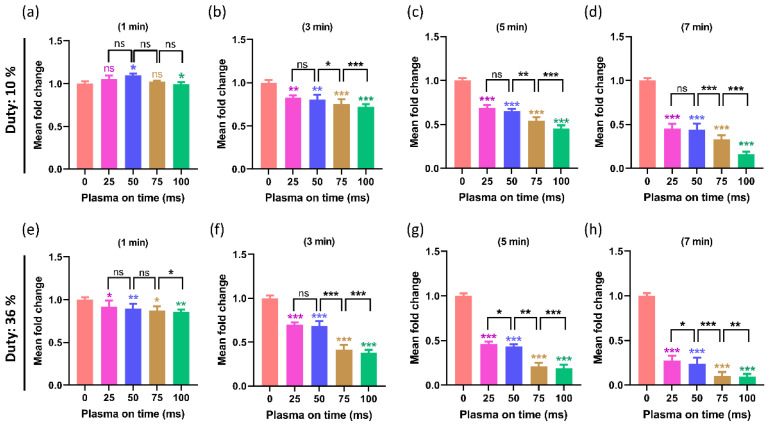
The U87-MG (brain cancer) cell line’s viability as measured 24 h after (PTM) treatment. The viability was observed under two different duty ratios: (**a**–**d**) when the duty ratio was fixed at 10% and (**e**–**h**) when the duty ratio was fixed at 36%. Only the plasma on-time varied for each duty ratio (25, 50, 75, and 100 ms), whereas the treatment time remained the same at 1, 3, 5, and 7 min. It was found that the viability declines between 25 ms and 50 ms plasma on time were non-significant in a 10% duty ratio but significantly decreased in a 36% ratio. It has been noted that, even when the duty ratio and treatment time were fixed, increasing the plasma on-time caused the viability of U87-MG cells to further decrease. The significance was calculated using Microsoft Excel (MS Office 365), while the differences between the treatment groups are indicated by * *p* < 0.05, ** *p* < 0.01, *** *p* < 0.001, ns—not significant.

The Alamar blue assay for measuring cell cytotoxicity was performed at two different duty ratios of 10% and 36%, and the results were shown in Figure 9. The U87-MG cell line is the most well known, and it is utilized to study brain cancers [41]. The viability of the U87-MG with a 10% duty ratio was shown in Figure 9a–d for treatment times of PTM that were 1, 3, 5, and 7 min, respectively. Similarly, Figure 9e–h indicate the viability of the U87-MG with a 36% duty ratio for treatment times of PTM, which were 1, 3, 5, and 7 min, respectively. In both duty ratio and treatment time, the treated groups show a decline in viability. Here, the work aims to find out the influence of plasma on-time on cell viability when treatment time and the duty ratio are fixed. The plasma on-time was changed to 25, 50, 75, and 100 ms in each treatment time. It is observed that the plasma on-time has a significant effect on viabilities and viability further decreased when plasma on time increased in both duty ratios. Interestingly, no difference in the viability decreased in 25 ms and 50 ms plasma on-time in a 10% duty ratio. These results were identical to the ROS (H_2_O_2_) content observed in PTM, as shown in Figure 8.

### 2.6. The Influence of the Plasma On-Time on Intracellular ATP Levels of U87-MG Cell Line

**Figure 10:**
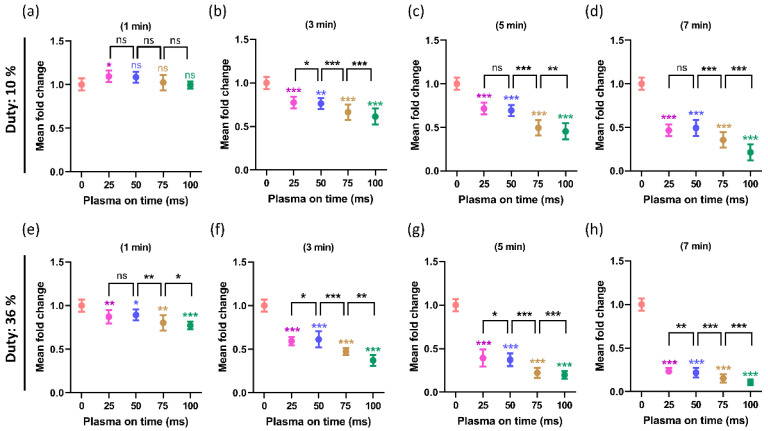
The intracellular ATP levels of the U87-MG cell line, which is observed 24 h after (PTM) treatment. The ATP was observed under two different duty ratios: (**a**–**d**) when the duty ratio was fixed at 10% and (**e**–**h**) when the duty ratio was fixed at 36%. It has been observed that increasing the plasma on-time results in a further decline in the ATP levels of U87-MG when the duty ratio and treatment time are fixed. The significance was calculated using Microsoft Excel (MS Office 365), while the differences between the treatment groups are indicated by * *p* < 0.05, ** *p* < 0.01, *** *p* < 0.001, ns—not significant.

When plasma was applied at two different duty ratios of 10% and 36%, the intercellular ATP levels of the U87-MG cell line were observed to support the viability and effect of the plasma over time. Figure 10a–d depict the ATP of the U87-MG with a 10% duty ratio for treatment times of 1, 3, 5, and 7 min, respectively. Similarly, Figure 10e–h show that the ATPs of the U87-MG with a 36% duty ratio for a treatment time of PTM were 1, 3, 5, and 7 min, respectively. The treated groups exhibit a decline in ATP levels during both duty ratio and treatment time. The purpose of this research is to determine how plasma on-time affects ATP levels under fixed treatment times of PTM (1, 3, 5, and 7 min) and duty ratio conditions. In each treatment time, the plasma time was modified by 25, 50, 75, and 100 ms. It has been found that the plasma on-time significantly affects ATP levels, which are further lowered when the plasma on-time is increased in both duty ratios. Interestingly, there was no difference in the rate of ATP decrease between plasmas at times of 25 ms and 50 ms at a 10% duty ratio. These findings are in good agreement with the ROS (H_2_O_2_) content and cell viability, as shown in Figure 8 and Figure 9.

## 3. Discussion

NTAPP has been used for many years to treat various cancers and tumors, and it has a variety of applications [38,42,43,44,45]. The ROS/RNS played a significant part in the biomedical applications of NTAPP, which is now widely acknowledged [29,38]. Variable anti-tumor efficacy is caused by feed gas admixtures and differences in the composition and quantity of plasma-generated ROS/RNS between devices [19]. Since these substances exhibit hormetic behavior in biology, it would make sense to investigate increased ROS/RNS exposure from cold physical plasmas as an anticancer agent [26]. Since cellular apoptosis is accelerated by NTAPP, the use of NTAPP has gained great interest and seems that it may be the next anticancer agent. Some studies have demonstrated that the impacts of NTAPP are significantly selective for cancer cells. The pH of the PTM remained unchanged after NTAPP, which is identical to our results [46,47]. The DMEM has the capacity to maintain the pH and temperature of the PTM [48]. Despite whether the effects of the plasma are direct or indirect, the right amount of time must be allowed for irradiation. It has been observed that, when the plasma exposure time is very long, both healthy and cancerous cells are damaged; however, when the exposure time is short, neither healthy nor cancerous cells are harmed. The therapeutic range of direct plasma treatment or PTM for applications must be optimized as a result [27,49]. ROS/RNS have therapeutic effects when administered to cells and tissues either directly from the plasma or by exposure to solutions that have already been plasma-treated. Optimization of the NTAPP treatment remained an active topic of research [50,51].

We kept the duty ratio and treatment time fixed while observing the impact of plasma on-time. The goal is to look into how a soft plasma jet’s electrical and optical properties are affected by plasma on-time. Additionally, the ROS/RNS concentrations are influenced by changing the plasma on-time and the physiochemical characteristics of the DMEM (PTM). By changing the plasma on-time, the electrical properties (current/voltage) do not change (Figure 2). The OES reveals that the longer plasma on-time may have resulted in an increase in ROS/RNS generation, which demonstrates an increase in the intensity (Figure 3). Sodium bicarbonate is the buffer that is most frequently used in mammalian cell culture [52]. The carbonic acid produced by the CO_2_ concentration makes the sodium bicarbonate buffered media more sensitive. The pH of the media can be maintained as long as the CO_2_ level is under control [53]. In this work, the pH of DMEM (PTM) was not decreased after NTAPP treatment due to this buffer (Figure 6).

The ROS/RNS levels in the PTM are directly correlated with both the ORP and EC [54]. In this study, the RNS/ROS delivered by the soft jet increased as NTAPP exposure to DMEM increased, increasing the EC and ORP of PTM (Figure 4 and Figure 5). It is interestingly noticed that the EC and ORP of the PTM drastically increased as plasma on-time continued to increase by 25, 50, 75, and 100 ms (Figure 4 and Figure 5). It is well known that the ROS/RNS plays a key role in the biomedical applications of NTAPP [23]. Various techniques were applied to increase the ROS/RNS levels within the therapeutic range of NTAPP [55,56]. It is intriguing to understand, from this work, that the ROS/RNS levels can be adjusted to meet needs by varying the plasma on-time within the fixed duty ratio and treatment time. By increasing the plasma on-time, the ROS/RNS levels (NOx and H_2_O_2_) were also noticeably elevated (Figure 7 and Figure 8). The results that are possible after a 3-min plasma treatment with a 10% duty ratio can be obtained after a 1-min plasma treatment with a 36% duty ratio, as shown in Figure 4, Figure 5 and Figure 7.

In many types of cancer, the ROS/RNS produced by NTAPP were directly responsible for cell apoptosis and death. Compared to normal cells, the U87-MG cells responded to NTAPP more strongly [57,58]. It has been demonstrated that NTAPP affects the morphology of cancer cells and triggers apoptosis while largely sparing healthy cells. ROS/RNS, which NTAPP can produce, may play important mechanistic roles in the treatment of cancer [57,58]. In this work, the ROS/RNS levels increased by increasing the plasma on-time (Figure 7 and Figure 8). It is important to investigate its impact on biological cells. It is interesting to observe that, when PTM was treated with different plasma on-times, it significantly influenced the viability and ATP levels of the U87-MG cell line (Figure 9 and Figure 10). The viability decreased in all PTM-treated groups, but the goal of this study was to closely examine how the viability and ATP levels of U87-MG cells changed when the plasma on-time was changed to 25, 50, 75, and 100 ms. It was found that, when the duty ratio and treatment time were maintained at a fixed value, the viability and ATP levels of the cells decreased, even more, when the plasma on-time was increased.

## 4. Materials and Methods

### 4.1. Experimental Setup with Measurement of Electrical and Optical Properties

A soft jet with air as the working gas was employed for the generation of the NTAPP. An experimental setup for the soft plasma jet is shown in Figure 1, which primarily consists of a power source, syringe needle electrode, cylindrical quartz tube, and cylindrical outer tube electrode. A 15-gauge syringe needle with an inner diameter of 1.37 mm and an outer diameter of 1.83 mm was used as a power electrode. With a flow rate of 1.5 lpm (liters per minute), the working gas air was passed only through an inner needle (hollow) power electrode. Between the electrodes, the quartz tube acted as a dielectric barrier to cause an electrical discharge. Electrodes that were separated from one another by a dielectric barrier produced plasma radicals [59]. The quartz tube with a 3.3 mm and 5 mm inner and outer diameter was covered by the needle electrode. The stainless-steel metal electrode that contains this quartz tube has inner and outer diameters of 6 mm and 10 mm, respectively. The stainless-steel metal electrode was connected to the ground. The gap between the grounded electrode (nozzle) and tip of syringe power electrodes was represented as d_g_ in Figure 1, which is adjusted at 1 mm distance. To operate the soft plasma jet, a 83 kHz sinusoidal wave was used by an inverter. The frequency of 83 kHz was obtained from measurements of the voltage and current waveforms shown in Figure 2b. To maintain the low temperature in NTAPP, a duty ratio was proposed [28], which is decided by the formula given as:

(1) $$Duty\;ratio=\frac{Plasma\;on\;time\;\left(ms\right)}{Plasma\;on\;time\;\left(ms\right)+Plasma\;off\;time\;\left(ms\right)}\times 100\;\left[\%\right]$$

The duty ratio was set to 10% and 36%, and plasma on-time was selected as 25, 50, 75, and 100 ms. Because this plasma jet is intended for biomedical use, we place a strong emphasis on temperature. Although it is obvious that the air plasma has a high temperature, we have managed to lower it by making the changes listed below. First, a duty ratio of 10% meant that, within 100 s of on-time and 900 s of off-time according to the above Equation (1), the off-time could limit the temperature increase by gas flow, without discharge. Second, the gap between the power and ground electrodes is only 1 mm, requiring less breakdown voltage while maintaining a gas flow rate of 1.5 lpm. These modifications enabled the production of low-temperature plasma. Furthermore, previous studies extensively utilized the soft plasma jet as a low-temperature plasma source [16,58,60]. At the end of the syringe and plasma generation region, the gap distance between the power and ground electrode is 1 mm. Without a quartz tube, only arc discharge occurs. The quartz tube is necessary for stable discharge and in order to achieve low-temperature plasma. Figure 1 illustrates the experimental setup in diagrammatic form. The plume was approximately 1 cm long, and 4 mm separated the soft jet’s nozzle from the surface of the Dulbecco’s Modified Eagle Medium (DMEM) cell culture media. Both fixed duty ratios of 10% and 36% were applied to the DMEM media to prepare PTM for 1, 3, 5, and 7 min for each plasma on-time (25, 50, 75, and 75 ms). In each condition, the DMEM volume remained at 3 mL. Two probes (indicated in Figure 1), a high-voltage probe (Tektronix P6015A), and a current probe (LeCroy CP030) were used to obtain the current–voltage waveform. Optical emission spectroscopy (OES) was also measured by using a spectrometer (HR4000+CG-UV-NIR, Ocean Optics, Inc., Orlando, FL, USA).

### 4.2. Properties of DMEM after Plasma Treatment

The pH and electric conductivity (EC) of the DMEM was measured by using a thermos scientific Orion multifunction benchtop meter. An amount of 3 mL of DMEM was treated by plasma and EC, and pH was immediately measured after treatment. The oxidation–reduction potential (ORP) of DMEM was also measured by using an ExStik meter (Extech, model: RE300, China) after treatment. At the two duty ratios of 10% and 36%, the pH, EC, and ORP were all measured at the treatment times (1, 3, 5, and 7 min). The plasma on-time was changed to 25, 50, 75, and 100 ms at each treatment time, and duty ratio and measurements of pH, EC, and ORP were obtained.

### 4.3. Estimation of Reactive Species

To measure the levels of H_2_O_2_ and NO_x_ in the media following NTAPP treatment, different duty ratios were utilized for the treatment of serum-free DMEM. For NO_x_ measurement, the Quantichrom Nitric oxide kit (BioAssay Systems D2NO-100) and H_2_O_2_ Quantichrom Peroxide assay kit (BioAssay Systems DIOX-250) were used. After the treatment in each selected condition, PTM was analyzed according to the kit manual protocol to quantify the ROS/RNS levels in control and treated groups.

### 4.4. Cell Viability Analysis

Alamar blue dye (DAL1025; Thermo Fisher Scientific, Waltham, MA, USA) was used to test U87-MG cells for metabolic viability. With a cell density of 1 × 10^4^ cells/mL, the cells were cultured in 96-well plates. Cells were treated with nonthermal plasma with varying duty ratios (10%, 36%) after 24 h incubation. With control and treated groups, experiments were carried out in at least three sets. Using a BioTek plate reader with an excitation wavelength of 540 nm and an emission wavelength of 600 nm, the fluorescence emission was measured in order to assess the Alamar blue dye’s conversion.

### 4.5. Intracellular ATP Measurement

Intracellular ATP level was measured inside cells after plasma treatment for more confirmation about cell death analysis. Cell Titer-Glo Assay, following the manufacturer’s instructions (Promega (cat no. G7572)), was used for intracellular ATP level measurement. For this, a 96-well plate with 1 × 10^4^ cells per well was used. Cells were added with an equivalent volume of prewarmed reagent and incubated for 1 h at 37 °C after 24 h of incubation following treatment. Utilizing a microplate reader, luminescence was assessed following incubation.

## 5. Conclusions

In this work, we sought to examine the impact of plasma on-times while maintaining a duty ratio and treatment time for the first time. For that, two different duty ratios of 10% and 36% were selected, and plasma on-time was altered as 25, 50, 75, and 100 ms. In each plasma on-time and duty ratio, the DMEM was treated for 1, 3, 5, and 7 min to prepare PTM. We have evaluated the electrical and optical properties of the soft jet, and OES shows the intensity of ROS/RNS increased by increasing plasma on-time. The pH and temperature of PTM remained unchanged or non-significant after treatment. It is also observed that the EC and ORP of the DMEM significantly increased by increasing the plasma on-time. Furthermore, the ROS/RNS levels in PTM were also investigated, and it was found that the plasma on-time directly affects the ROS/RNS content in PTM and increased by increasing the on-time of plasma when the duty ratio and treatment time were fixed. Finally, the PTM treated with different plasma on-time was implemented on brain cancer cell line U87-MG to observe the cell viability and ATP levels. Interestingly, we observed that, by extending the plasma on-time by 25, 50, 75, and 100 ms, the levels of ROS/RNS significantly rose in PTM and had a significant impact on the viability and ATP levels of the brain cancer U87-MG cell line. It was also noticed that results that are possible after a 3-min plasma treatment with a 10% duty ratio can be obtained after a 1-min plasma treatment. It is intriguing to understand, from this work, that the ROS/RNS levels can be adjusted to meet needs by varying the plasma on-time within the fixed duty ratio and treatment time. The results of this study provide a significant indication of advancement by introducing the optimization of plasma on-time to increase the efficacy of the soft plasma jet for biomedical applications.

## Figures and Tables

**Table 1:** The plasma on/off time when the duty ratio was fixed at 10% and 36%.

**Duty Ratio 10%/36%** **Plasma On-Time**	**Duty Ratio 10%** **Plasma Off-Time**	**Duty Ratio 36%** **Plasma Off-Time**
25 ms	225 ms	44 ms
50 ms	450 ms	89 ms
75 ms	675 ms	133 ms
100 ms	900 ms	178 ms

## Data Availability

The data that support the findings of this study are available from the corresponding author upon reasonable request.

## Supplementary Materials

The following supporting information can be downloaded at: https://www.mdpi.com/article/10.3390/ijms24065289/s1.
