# Supplementary material for: Effect of Plasma On-Time with a Fixed Duty Ratio on Reactive Species in Plasma-Treated Medium and Its Significance in Biological Applications
Source: Int J Mol Sci. 2023 Mar 9;24(6):5289. doi: 10.3390/ijms24065289 (PMC10049170; doi:10.3390/ijms24065289)
Supplement: Supplementary file 1 [file ijms-24-05289-s001.zip › ijms-2140682-supplementary.pdf]

Effect of plasma on-time with a fixed duty ratio on reactive species in plasma treated medium and their significance in biological applications

Sohail Mumtaz, Juie Nahushkumar Rana, Jun Sup Lim, Rida Javed, Eun Ha Choi, and Ihn Han*

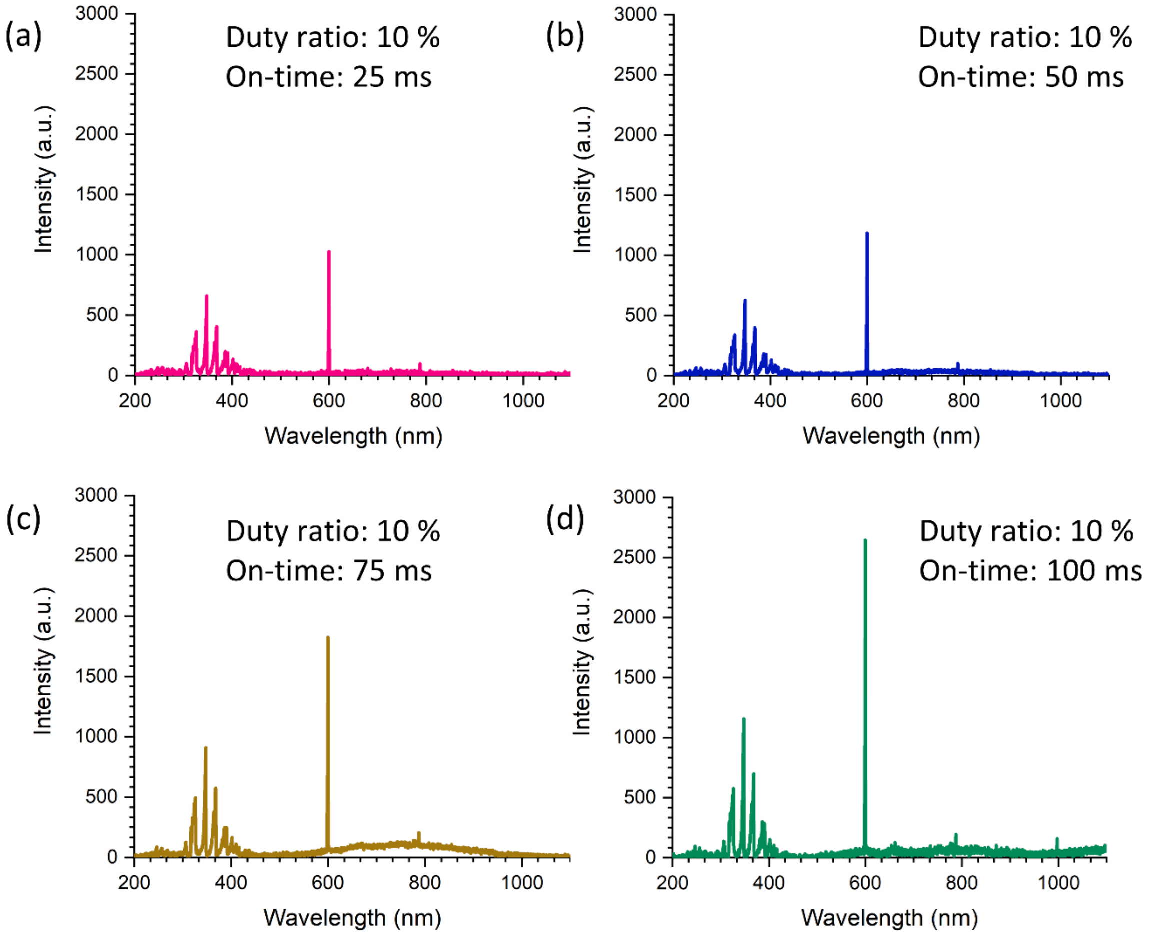

Figure S1. The OES at various plasma on-times of (a) 25 ms, (b) 50 ms, (c) 75 ms, and (d) 100 ms when the duty ratio was fixed at 10%.

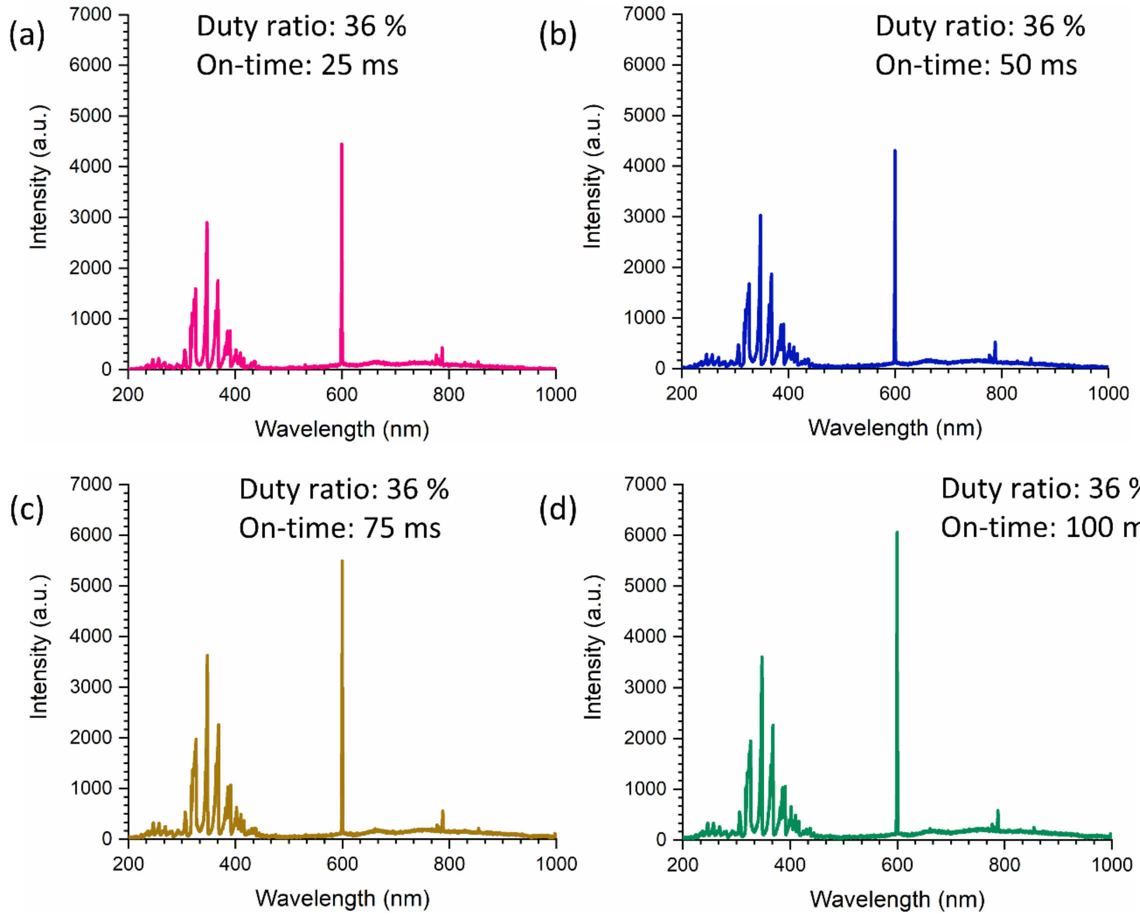

Figure S2. The OES at various plasma on-times of (a) 25 ms, (b) 50 ms, (c) 75 ms, (d) and 100 ms when the duty ratio was fixed at 36%.

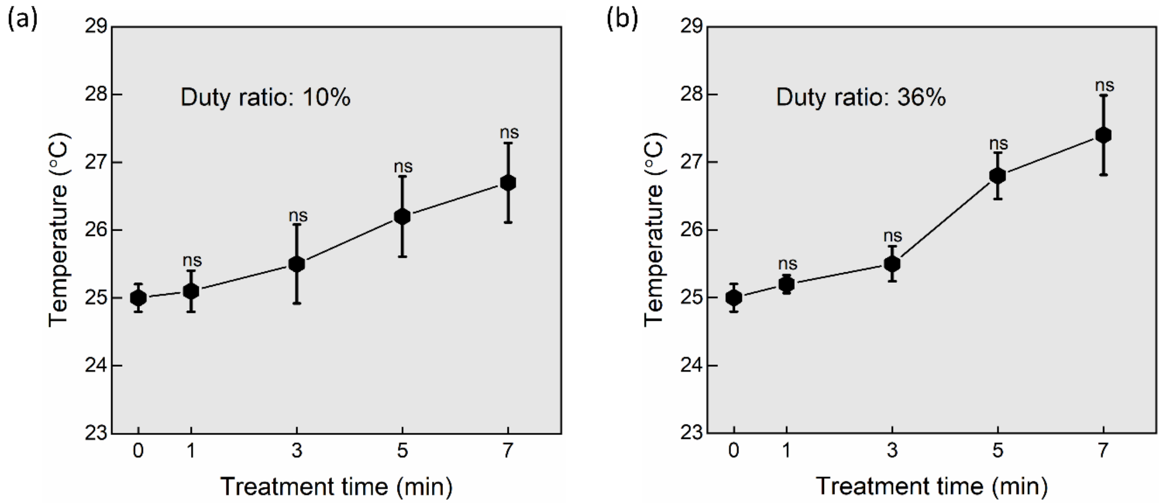

Figure S3. The temperature of the PTM (DMEM) after plasma treatment. (a) The temperature of DMEM after treatment when duty ratio is 10%. (b) The temperature of DMEM after treatment when duty ratio is 36%. The temperature of the PTM has a slight increase (10%: ~ 1 – 1.5 °C, 36%: 1 – 3 °C) at treatment period of 5 min and 7 min, which is nonsignificant compared to control group.
